# Can platelet-rich plasma coating improve polypropylene mesh integration? An immunohistochemical analysis in rabbits

**DOI:** 10.1590/S1677-5538.IBJU.2020.0017

**Published:** 2021-02-03

**Authors:** Fernando Goulart Fernandes Dias, Sílvio Henrique Maia de Almeida, Wagner Fávaro, Paulo Latuf, Cássio L. Z. Riccetto

**Affiliations:** 1 Universidade Estadual de Campinas - UNICAMP Departamento de Urologia Campinas SP Brasil Departamento de Urologia, Universidade Estadual de Campinas - UNICAMP, Campinas, SP, Brasil.; 2 Universidade Estadual de Londrina - UEL Departamento de Cirurgia Londrina PR Brasil Departamento de Cirurgia, Universidade Estadual de Londrina - UEL, Londrina, PR, Brasil.; 3 Universidade Estadual de Campinas - UNICAMP Departamento de Biologia Estrutural e Funcional Campinas SP Brasil Departamento de Biologia Estrutural e Funcional, Universidade Estadual de Campinas - UNICAMP, Campinas, SP, Brasil.; 4 Universidade Estadual de Campinas - UNICAMP Centro de Investigação em Pediatria (CIPED FCM) Campinas SP Brasil Centro de Investigação em Pediatria (CIPED FCM), Universidade Estadual de Campinas - UNICAMP, Campinas, SP, Brasil.

**Keywords:** Urinary Incontinence, Female, Tissue Engineering, Suburethral Slings

## Abstract

**Purpose:**

Despite high success rates in the treatment of urinary incontinence, complications related to the use of polypropylene (PP) meshes are still a concern, especially in vaginal prolapses surgeries. The objective of this study was to assess the effect of autologous platelet-rich plasma (PRP) coating on the integration of PP meshes implanted in the vaginal submucosa of rabbits.

**Materials and Methods:**

Thirty adult New Zealand rabbits were randomly divided into two groups (n=15): PP, implanted with conventional PP meshes; and PRP, implanted with autologous PRP coated PP meshes. Animals in both groups (n=5) were euthanized at 7, 30 and 90 days postoperatively, the vaginas extracted and sent to immunohistochemical analysis for the assessment of the pro-inflammatory agent TNF-α, anti-inflammatory agents TGF-β and IL-13, collagen metabolism marker MMP-2, and angiogenesis marker CD-31. AxioVision™ image analysis was used for the calculation of the immunoreactive area and density. Statistical analysis was performed with ANOVA followed by Tukey test (p <0.05).

**Results:**

Animals in the PRP group showed significantly increased expression of the angiogenesis agent CD-31 at all experimental times when compared to the PP group (p <0.0001). However, no differences concerning the expression of the other markers were observed between the groups.

**Conclusion:**

The addition of autologous PRP gel to PP meshes can be simply and safely achieved and seems to have a positive effect on implantation site angiogenesis. Further investigations are required to ascertain PPR coated meshes clinical efficacy in prolapses and stress urinary incontinence surgeries.

## INTRODUCTION

With the rapid aging of the world's population, pelvic organ prolapses (POP) and stress urinary incontinence (SUI) have become common health problems among women, with serious effects on their health, as well as their personal, sexual, and professional life (1).

Polypropylene (PP) meshes have been used for decades, and are considered the gold standard in urogynecology surgeries. It is a simple technique with higher success rates than traditional tissue self-repair. However, after the American Food and Drug Administration (FDA) reported on more than 3500 unexpected and severe adverse events in 2008 and 2011, the transvaginal placement of surgical meshes has become a source of concern worldwide (2).

While many factors may influence complications rates, such as surgeon experience and the correct indication for implantation and dissection techniques, mesh features can also represent a determinant factor (3). There is evidence that changes to mesh surface can influence tissue response in vivo (4). Although several different types of biomaterials have been tested for mesh coating purposes, no consistent results have been reported so far (5).

Platelet-rich plasma (PRP) is a platelet concentrate 3-5 times higher than the normal count, obtained through centrifugation of a sample of the patient's own blood. Since the 1990s PRP has been used in several different fields of regenerative medicine (6, 7). The potential therapeutic effect of PRP is based on its ability to improve tissue regeneration by releasing growth factors present in platelet alpha granules (8). Considering its effects on wound healing hemostasis, proliferative, and remodeling phases, PRP could be considered as a coating agent for PP meshes in urogynecology and pelvic reconstructive surgeries. Nevertheless, animal studies have been suggested in order to better ascertain the host's response to implants coated with PRP (9).

Therefore, the aim of this study was to evaluate the effect of autologous PRP coating on the integration of monofilament PP meshes in comparison with conventional uncoated meshes implanted in the vaginal wall of rabbits. We hypothesized that PRP coated meshes would result in improved immunoinflammatory response, collagen metabolism, and angiogenesis when compared to uncoated meshes.

## MATERIALS AND METHODS

### Ethical aspects

This preclinical animal study was performed after approval by the Ethics Committee for Animal Experiments of the State University of Londrina, Brazil (CEEA-IB-UEL, no. 12071.2013.21), and all procedures were conducted according to the ethical principles adopted by the Brazilian College of Animal Experimentation (COBEA).

### Sample

The sample consisted of thirty white female New Zealand rabbits (Oryctolagus cuniculus), specially bred for research purposes, aged between 3 and 6 months and weighing between 2.5 and 4kg (mean 3.2kg).

At the moment of the surgery, the rabbits were randomly allocated (n=15) either to the PP group (control), which received a conventional 10 × 10mm monofilament PP mesh with pores measuring 1500pm, or to the PRP group (test) - implanted with same mesh coated with autologous PRP gel.

### PPR gel preparation

Autologous PRP in a gel form was prepared according to the protocol described by Anitua et al. (10). After anesthetic induction with ketamine 40mg/ kg and xylazine 3mg/kg, 5mL of blood were collected from each animal through cardiac puncture. The blood was transferred to a 1.8mL sterile polypropylene tube containing 0.10mL of 3.2% sodium citrate. After homogenization, the tube was centrifuged at 1200rpm for 10 min., and 1.2mL of the plasma was transferred into a sterile plastic Eppendorf tube, which was centrifuged at 2000rpm for another 10 min. The supernatant was discarded, and 5μL of 10% sterile calcium gluconate was added to the remaining material and homogenized for 15 min in order to acquire a gel consistency. Platelet counts were conducted in 25% of randomly chosen gel samples to confirm platelet concentration. An average platelet count 4.5 times higher than that normally found in peripheral blood (360.000 vs. 80.000/μL) was obtained. The gel was carefully spread on the PP mesh, ensuring that the entire length and the interstices between the mesh pores were filled.

### Mesh implantation

Once adequately anesthetized and with the animals in a horizontal dorsal decubitus position, vagina antisepsis was conducted with 10% iodopovidone alcohol solution. A 2cm transverse incision was performed at the introitus in the posterior vaginal wall, and the epithelial layer was laterally and longitudinally dissected to a distance of approximately 2cm to reveal the rectovaginal fascia. After exposing the underlying fascial tissue, the implant was inserted without fixation to prevent tissue response. The PP mesh was implanted in a standardized manner, between the hypodermis and the fascia of the abdominal muscles.

After the implantation, the vaginal mucosa was sutured with a single stitch using a 3.0 nylon thread. Postoperative analgesia was performed orally with tramadol solution 1mg/kg, at every 6 hours for 48 hours, and antibiotic prophylaxis with a single injection of penicillin benzathine 40.000IU/kg.

Animals from both groups (n=5) were euthanized at 7, 30 and 90 days postoperatively, and an en bloc excision containing all layers of the vaginal wall (skin, subcutaneous, mesh, and muscle aponeurosis) was performed. The samples were placed in a container with 10% formaldehyde for 48 hours, after which they were transferred to a 70% alcohol solution. Then, tissue specimens were fixed in 10% formalin, embedded in paraffin, sectioned and mounted on numbered slides. Five slides containing 3 sections of the surgical specimen were mounted for each animal.

Immunohistochemical sample preparation Based on the literature (11–14), immunohistochemical analysis was conducted by using rabbit-specific antibodies in order to assess: (a) immunoinflammatory response, evaluated by the proinflammatory agent tumor necrosis factor receptor alpha (TNF-α), and the anti-inflammatory agents transforming growth factor beta (TGF-β) and interleukin-13 (IL-13); (b) collagen metabolism, evaluated by matrix metalloproteinase-2 (MMP-2); and (c) angiogenesis, assessed by cluster of differentiation-31 (CD-31).

Sections were incubated at room temperature for 30 min and at 8°C overnight with mouse monoclonal antibody to Anti-IL13 receptor alpha 2 (polyclonal, Abcam 55275 diluted at 1: 1000); TNF-α receptor-1 (polyclonal, Abcam 19139, diluted at 1: 1000); TGF-β 1 (polyclonal, Abcam 9758, diluted at 1: 800); MMP-2 (clone [4D3], Abcam 2462, diluted at 1: 250); CD-31 (clone JC 70A, Abcam 9498 diluted at 1: 500). Antigen-antibody binding was observed using an advanced detection system (Dako EnVision™), and immunostaining achieved using diaminobenzidine (DAB). Positive internal and external controls included tissue samples as indicated on the datasheet of the antibodies used. The same tissue samples used for positive controls were also used as negative controls by omitting the primary antibody.

### Immunohistochemical analysis

Histological evaluations were conducted with a Zeiss Primo Star™ microscope connected to a Zeiss AxioCam ICC 1™ camera (Carl Zeiss Microscopy, Jena, Germany). Only one single experienced pathologist blinded to the groups and times evaluated all specimens. The slide for each animal, containing three surgical specimens, was analyzed under X 20 magnification, and three fields of each slide were randomly selected for subsequent image acquisition.

**Figure 1:**
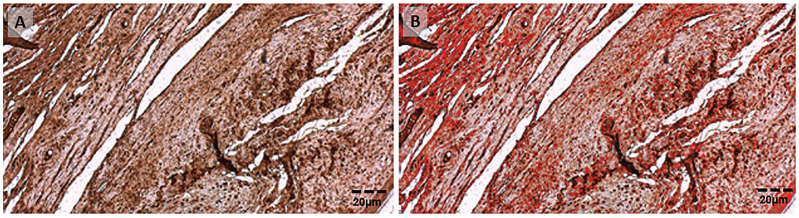
A) immunoreactive expression of MMP2. B) A special feature of the AxioVision™ Microscope V 4.8.0.0 Software highlighting the reactive area in red for the automatic calculation of the reactive area (45.1%), and density (153.9). (20x magnification). Scale bars=20pm for A and B.

Objective analysis of the immunoreactive expression was performed with the use of an image analysis program (AxioVision™ Microscope V 4.8.0.0 Software). After field definition, the program automatically calculated the extension of the immunoreactive area and the density of the specific immunoreactive expression (Figure-1). For each variable, the final immunoreactive area and density were obtained by analyzing each of the three selected fields and calculating the means.

#### Statistical Analysis

Mean immunoreactive area and density were statistically analyzed using repeated measures ANOVA followed by Tukey post hoc test to compare groups and experimental times at a 95% level of significance (p <0.05).

## RESULTS

All the animals survived the surgical procedures without any intraoperative or postoperative complications. Table-1 illustrates the mean immunoreactive area and density values obtained for the studied variables. Table-2 illustrates the statistical results (p values) comparing groups and experimental times.

**Table 1:** Mean area and density of IL-13, TGF-β, TFN-α, MMP-2, and CD-31 expression in the PP (polypropylene mesh) and PRP (polypropylene mesh + platelet-rich plasma) groups at the different experimental times.

Variables	Groups	7 days		30 days		90 days	
		Area (%)	Density	Area (%)	Density	Area (%)	Density
IL-13	PP	22.34	87.28	23.13	87.88	19.24	87.54
	PRP	21.56	87.48	18.51	86.82	16.53	87.17
TGF-β	PP	21.68	107.19	30.60	108.46	26.11	107.26
	PRP	23.93	106.64	27.54	107.39	20.42	106.49
TFN-α	PP	10.87	60.26	7.66	60.64	8.66	61.24
	PRP	6.45	60.19	7.34	60.51	8.76	60.43
MMP-2	PP	39.76	55.93	40.67	61.82	36.52	58.52
	PRP	44.34	59.82	44.93	57.94	28.27	54.99
CD-31	PP	2.41	108.18	1.11	104.72	1.65	107.28
	PRP	6.34	108.70	4.84	104.78	5.83	107.93

**Table 2:** Statistical results (p values) comparing times (7, 30 and 90 days) and PP (polypropylene mesh) and PRP (polypropylene mesh + platelet-rich plasma) groups.

Markers	Immunoreactive	Comparison among	Comparison between
		Times (p)	Groups (p)
IL-13	Area	0.0051^a^	0.0672
	Density	0.9888	0.0956
TGF-β	Area	0.0778	0.5656
	Density	0.2951	0.0945
TFN-α	Area	0.5839	0.1160
	Density	0.0967	0.1930
MMP-2	Area	0.0223^b^	0.9462
	Density	0.3912	0.4527
CD-31	Area	0.0420^c^	<0.0001^d^
	Density	0.0006^e^	0.3102

a 7≠90, 30≠90

b 7≠90, 30≠90

c 7≠30

d PP<PRP

e 7≠30, 90≠30

No significant differences in the immunoreactive area or density were observed between groups with regards to the anti-inflammatory response (IL-13 and TGF-β). However, when the experimental times were compared, a significant decrease in the immunoreactive area of IL-13 was observed in both groups at 90 days when compared to the other times (p=0.0051).

The pro-inflammatory response (TNF-α) presented no significant differences in mean immunoreactive area among times (p=0.5839) or between groups (p=0.1160), neither in mean density among times (p=0.0967) or between groups (p=0.1930).

Collagen metabolism (MMP-2) did not present significant differences between groups in terms of area (p=0.9462) or density (p=0.4527). However, similarly to IL-13, a significant decrease in the area was observed in both groups at 90 days in relation to the other times (p=0.0223).

**Figure 2:**
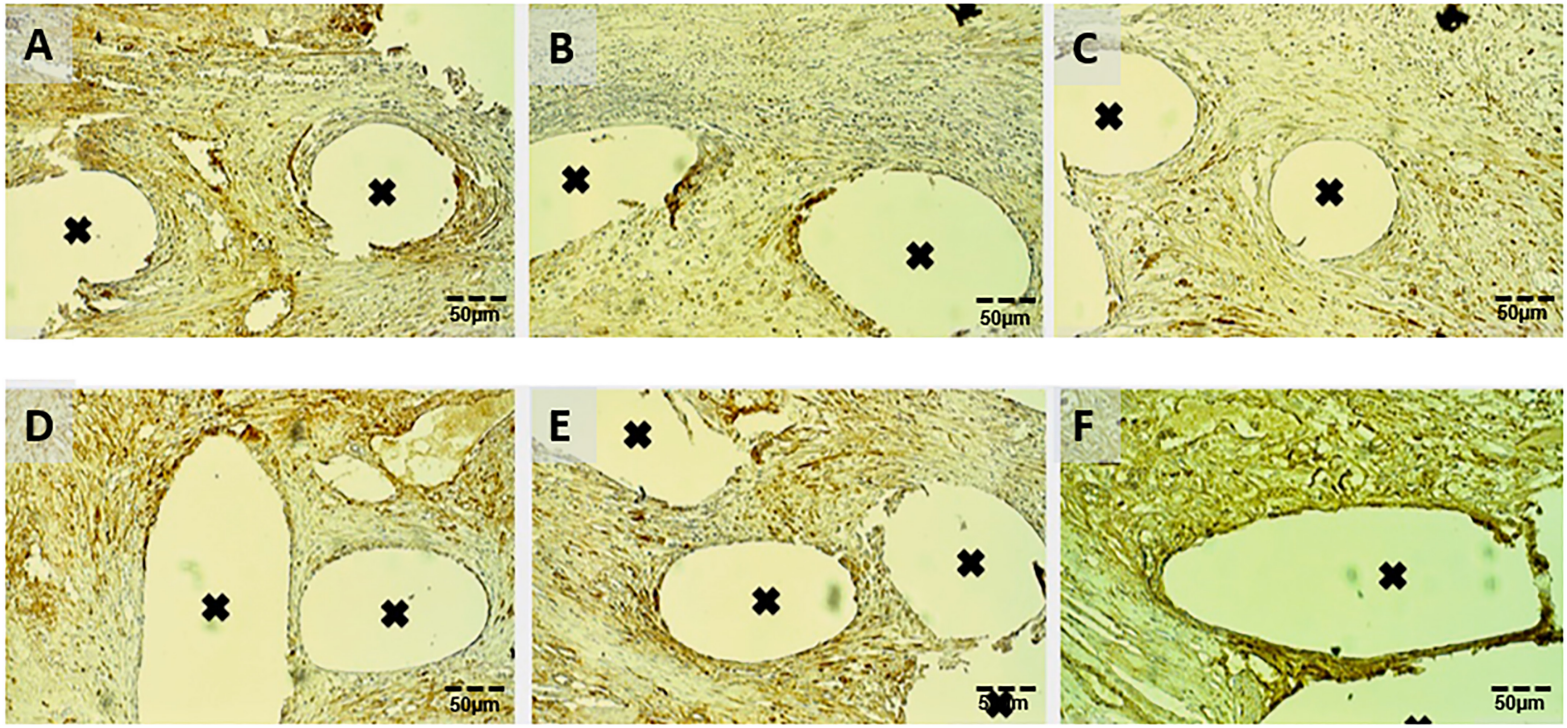
Voids marked with a black cross were previously occupied by mesh filaments, while the brownish areas in the surrounding tissue is the immunoreactive expression of CD-31. A, B and C) PP group at 7, 30 and 90 days, respectively. D, E, and F) PRP group at 7, 30, and 90 days, respectively. Immunoreactive areas A=2.4%; B=1.1%; C=1.6%; D=6.3; E=4.1%; F=5.8% (20x magnification). Scale bars=50μm for (A-f).

The angiogenesis expression agent CD-31 demonstrated significant differences between groups, with the PRP group presenting a significantly larger immunoreactive area when compared to the PP group at all experimental times (p=<0.0001; Table-2; Figure-2). At 30 days, both groups presented a significantly decreased area (p=0.0420) and density (p=0.0006) when compared to the other times.

## DISCUSSION

In this preclinical study, the integration of PRP coated monofilament PP meshes was assessed in comparison with uncoated meshes implanted in the vaginal wall of rabbits. The results demonstrated a significantly higher expression of the angiogenesis marker CD-31 in the PRP group when compared with the PP group. However, no statistical differences were observed between groups in the expression of the other studied markers. Hence, the working hypothesis was only partially supported by the results.

Some studies have been recently conducted in an attempt to develop optimized surgical meshes that can permit the transmigration of beneficial host cells, avoiding extensive local inflammation and improving biocompatibility. As a result, changes in meshe's weight and pore size, different types of polymers, such as polyvinylidene fluoride (PVDF), as well as surface coating (collagen, titanium or absorbable polymers) have been tested (15). Among the possible candidates for mesh coating, platelet derivatives have emerged as an interesting alternative. Apart from its critical role in wound healing hemostasis, proliferative and remodeling phases (16), a previous in vitro study suggested that plasma coated meshes could perform better than other types of mesh in terms of biocompatibility (5).

Autologous PRP can be simply and quickly prepared from the patient's own blood, eliminating the risk of infection transmission and immunogenic responses, observed with allograft and xenograft preparations (17). The cost involved in its preparation is minimal and, to date, no evidence of systemic or carcinogenic effects have been reported (17). Most of the side effects described are local, venipuncture-related during blood collection, rarely resulting in scarring or calcification at application sites (17). The application of PRP in the management of soft tissue injuries has only recently been investigated, and the results reported have been controversial. In some animal studies, PRP has demonstrated clear benefits in terms of accelerated healing, whereas others have failed to show any significant biomechanical benefits (18). This discrepancy may be explained by the fact that large multicenter randomized trials with representative sample sizes are yet to be conducted to validate the therapy.

The use of PRP in urogynecology is still incipient with just a few published attempts. The vaginal implant site is unique due to the specific vaginal microenvironment, dynamics, biochemical exchange, and immunological response. Hence, results obtained in other medical areas, such as inguinal hernia surgery, cannot be directly extrapolated. The endogenous microflora in the vagina creates a hostile environment to vaginally implanted graft materials, leading to autolysis or rejection. Therefore, graft material investigations in a vaginal model are essential to shed some light on how the vaginal compartment integrates, rejects, or even changes graft materials. Rabbits have a large vaginal cavity with bacterial flora similar to the human vagina (19). In a previous study conducted by our research group using rabbits, the effect of PRP added to PP meshes was associated with increased concentrations of collagen I and III seven days postoperatively (20).

The first study in which plasma coated meshes were used in urogynecology in humans was performed with 20 patients indicated for SUI and POP repair. The authors concluded that the procedure was safe, easy to execute, with good functional outcomes, with no severe complications (21). Subsequently, some studies have also demonstrated PRP safety in gynecological surgeries, with no apparent side effects (10). Recently, using platelet-rich fibrin in site-specific prolapse corrections, Gorlero et al. obtained an 80% success rate and a 100% improvement in prolapse symptoms at 24 months (22). In contrast, in a clinical study with a small number of patients, Einarsson et al. revealed that PRP use during colporrhaphy did not seem to increase collagen content or durability of the surgical repair (23). Nonetheless, PRP has been recently proposed as an alternative therapy for the treatment of some complications in urogynecology, such as vesicovaginal fistula (24), and vaginal mesh exposure after abdominal sacral colpopexy (2).

Our findings showed no significant effect of PRP coated meshes on the expression of inflammation and collagen metabolism markers after implant. However, a significant decrease in MMP-2 and IL-13 immunoreactive area was observed at 90 days postoperatively in both groups, indicating that these markers tend to have an attenuated role in late mesh integration.

On the other hand, animals in the PRP group demonstrated a significantly higher expression of the angiogenesis marker CD-31 at all times when compared to the PP group, indicating an improved effect on neovasculari zation after mesh implant. The enhanced angiogenesis activity can be explained by the autologous growth factors (including PDGF, VEGF, TGF-β, and EGF) found in PRP, and the way they are released by hydrogels (25). Angiogenesis is critical to wound healing and tissue regeneration, providing a mechanism for new healing factors to be brought into the wound. The slow release of angiogenic growth factors in the PRP gel may contribute to enhanced neovascularization, stabilization of newly formed vessels, and improved wound healing. However, no clear evidence is yet available to support that increased angiogenesis can reduce mesh-related complications.

The results of the present study must be interpreted cautiously. The rational use of animals for research purposes imposes a limit on the sample size. Another important limitation concerns the very small number of studies assessing PRP using immunohistochemical analysis, preventing direct comparisons. The choice of reagents is also limited by the lack of immu- nohistochemical antibodies present in the reactivity of rabbits. Moreover, the different PRP preparation techniques found in the literature with different blood volumes, platelet separation systems, activating agents, forms of application, and platelet concentrations, also impair proper comparisons (19). However, despite these limitations, our findings seem to indicate that PRP coated PP meshes may become a feasible and beneficial alternative in reconstructive surgeries in the foreseeable future.

## CONCLUSION

Based on the results and limitations of the present study, the implantation of PRP coated meshes in a vaginal model in rabbits was shown to be a simple, safe and low cost procedure, which may result in some benefit to the patient. From an immunohistochemical point of view, the procedure seems to have a positive effect on implantation site angiogenesis. Further studies using different PRP preparation methods and forms of delivery are required to clarify the real benefits and clinical applicability of PRP coated meshes in prolapses and stress urinary incontinence surgeries.
